# The NMDA receptor antagonist MK-801 fails to impair long-term recognition memory in mice when the state-dependency of memory is controlled

**DOI:** 10.1016/j.nlm.2019.03.006

**Published:** 2019-05

**Authors:** Michele Chan, Joseph M. Austen, Madeline J. Eacott, Alexander Easton, David J. Sanderson

**Affiliations:** a Department of Psychology, Durham University, South Road, Durham DH1 3LE, UK; b Centre for Learning and Memory Processes, Durham University, Durham, UK; c Department of Psychology, University of Essex, Colchester, Essex CO4 3SQ, UK^1^; d Department of Psychology, Koç University, Rumelifeneri yolu, Sarıyer, 34450, Istanbul, Turkey^1^

**Keywords:** Memory, Habituation, NMDA receptors, Mice, MK-801

## Abstract

NMDA receptor-dependent synaptic plasticity has been proposed to be important for encoding of memories. Consistent with this hypothesis, the non-competitive NMDA receptor antagonist, MK-801, has been found to impair performance on tests of memory. Interpretation of some of these findings has, however, been complicated by the fact that the drug-state of animals has differed during encoding and tests of memory. Therefore, it is possible that MK-801 may result in state-dependent retrieval or expression of memory rather than actually impairing encoding itself. We tested this hypothesis in mice using tests of object recognition memory with a 24-hour delay between the encoding and test phase. Mice received injections of either vehicle or MK-801 prior to the encoding phase and the test phase. In Experiment 1, a low dose of MK-801 (0.01 mg/kg) impaired performance when the drug-state (vehicle or MK-801) of mice changed between encoding and test, but there was no significant effect of MK-801 on encoding. In Experiment 2, a higher dose of MK-801 (0.1 mg/kg) failed to impair object recognition memory when mice received the drug prior to both encoding and test compared to mice that received vehicle. MK-801 did not affect object exploration, but it did induce locomotor hyperactivity at the higher dose. These results suggest that some previous demonstrations of MK-801 effects may reflect a failure to express or retrieve memory due to the state-dependency of memory rather than impaired encoding of memory.

## Highlights

• The role of NMDA receptors in encoding of object memory was assessed.
• A retention interval of 24 h was used.
• When state-dependency was controlled MK-801 failed to impair memory.

## 1 Introduction

N-methyl-D-aspartate (NMDA) receptors, due to their role in the putative learning mechanism long-term potentiation (Bliss & Collingridge, 1993), have been proposed to be important for encoding of memories (Riedel, Platt, & Micheau, 2003). Evidence for the role of NMDA receptors in encoding has, in part, come from studies that have examined the effects of MK-801 on tests of memory. MK-801 (dizocilpine) is a non-competitive and selective NMDA antagonist that has a high affinity towards NMDA receptors (Wong et al., 1986) and acts by binding to NMDA receptors and blocking the channel pore in a use-dependent manner. MK-801 has been shown to impair short-term spontaneous object recognition memory (King et al., 2004, van der Staay et al., 2011), a form of memory reliant on the perirhinal cortex (Ennaceur, Neave, & Aggleton, 1996). MK-801 has been suggested to have the greatest effect when the drug is administered before the encoding phase (de Lima et al., 2005, Nilsson et al., 2007) and doses as low as 0.01 mg/kg have been found to impair performance (de Lima et al., 2005). The effect of the drug is less clear, however, when given after the encoding phase or prior to the test, with some studies (de Lima et al., 2005, Pichat et al., 2007) reporting impairment of performance whilst other studies reported facilitating effects of the drug (Nilsson et al., 2007) on objection recognition memory.

Studies that have attempted to dissociate the effects of MK-801 on encoding and retrieval of memory have, typically, separated the encoding and retrieval phases by a period of time after which it is unlikely that the drug will still be effective (e.g., 24 h). A problem with this approach is that in order to isolate the effects of a drug on a particular stage of a memory procedure, animals are tested in a drug-state that differs from the drug-state during encoding. Therefore, if behavioral expression or retrieval of memory is dependent on animals being in the same state as during encoding then a change of state will impair performance (Girden & Culler, 1937). Previous research that has examined the effect of MK-801 on long-term object recognition, in which the exposure and test phases are separated by a long period of time, have failed to control for the state-dependency of memory. de Lima et al. (2005) gave doses of 0.01 and 0.1 mg/kg MK-801 20 min before the encoding phase and rats were tested 24 h later. Reductions in performance were found with both doses. This raises the possibility that MK-801 may not have impaired object memory encoding itself, but instead affected performance through altering the drug-state of animals. Consistent with this possibility, MK-801 has been found to have state-dependent effects on performance on other learning and memory procedures such as fear induced passive avoidance (Ceretta et al., 2008, Flint et al., 2013, Harrod et al., 2001) and appetitively rewarded instrumental responding (Jackson, Koek, & Colpaert, 1992).

It is important to note that state-dependency of object recognition memory performance is less likely to be an issue when memory is tested after a relatively short interval, because the encoding and retrieval stages of the test are likely to be conducted under the same drug-state. For example, in the study by van der Staay et al. (2011) MK-801 was given 30 min before the encoding phase and rats were tested after a one hour retention interval (see also King et al., 2004, in which MK801 was given 40 min before encoding and the retention interval was two hours). Therefore, the significant effect of MK-801 on performance in this experiment is unlikely to be due to a change in drug-state after this short period of time. State-dependency is more likely to be an issue when the interval between encoding and test is long enough to exceed the duration of the effect of drug.

In the present study, we tested whether MK-801 induced a state that affected performance of memory by testing long-term object recognition memory in mice. Mice were tested using a procedure that allows for multiple tests of object recognition memory, which increases the overall sensitivity of the measure (Ameen-Ali et al., 2012, Chan et al., 2018). Mice were exposed to eight different objects on day 1 and then on day 2 received a series of novelty preference tests, one for each of the eight familiar objects, in which they were allowed to explore a familiar object and a novel object. A preference for exploring the novel object over the familiar object demonstrates stimulus-specific habituation of exploratory behavior towards the familiar object as a result of a memory for the familiar object. In Experiment 1, we examined the effect of a low dose of MK-801 (0.01 mg/kg) on state-dependent, long-term object recognition memory. This dose has previously been reported to be sufficient to disrupt performance when administered prior to the encoding phase of an object recognition memory test in rats (de Lima et al., 2005). Mice received either MK-801 or vehicle prior to the exposure phase on day one and then received either the same substance or the different substance prior to the test on day two. Therefore, the factors of drug-state at exposure and whether the drug-state at test matched that of exposure were manipulated in a factorial manner. In Experiment 2, we tested the effect of a higher dose of MK-801 (0.1 mg/kg). Mice received either MK-801 prior to both exposure and test, or vehicle prior to both phases. Therefore, the drug-state at both phases was the same and so performance in the test phase cannot reflect the effect of a switch between drug-states. In addition to object exploration, we also measured path lengths during the procedure in order to gauge whether the doses of MK-801 caused hyper-locomotor activity.

## 2 Materials and methods

### 2.1 Subjects

Thirty-two female C57BL/6J mice, bred in Life Sciences Support Unit at Durham University, were used in Experiments 1 and 2. Mice were approximately 13 weeks old at the start of testing and their free-feeding weights ranged between 15.9 and 22.6 g (mean = 19.1 g). The animals were housed in groups of four under diurnal conditions (lights on: 7:00 am–7:00 pm). Prior to the start of the experiment, the weights of the mice were reduced by being placed on a restricted diet. Mice were then maintained at 85% of their free-feeding weights throughout the experiment. Mice had ad libitum access to water in their home cages. All procedures were in accordance with the United Kingdom Animals Scientific Procedures Act (1986); under project license number PPL 70/7785.

### 2.2 Drugs and injections

(+)-MK-801 hydrogen maleate (Sigma-Aldrich, UK) was dissolved in saline (0.9% NaCl solution). Mice were injected intraperitoneally with MK-801 or vehicle 30 min before the exposure phase and test phase. Doses of 0.01 mg/kg and 0.1 mg/kg MK-801, given in a volume of 10 mL/kg, were used in Experiments 1 and 2, respectively.

### 2.3 Apparatus

A rectangular arena (50 cm × 42 cm × 20 cm) divided into a holding arena and an object arena was used (see Chan et al., 2018, Fig. 1). Mice could move between the arenas through three guillotine doors controlled by the experimenter. There was a central door (15 cm wide) that allowed mice to move from the holding arena to the object arena, and two side doors (10 cm wide) that allowed mice to return from the object arena back to the holding arena. Objects were placed at the top-left and top-right corner of the object arena approximately 3 cm from the walls. Two food wells, one in each of the holding and object arenas, were located in the middle of the end walls of the apparatus. The apparatus was made of 10 mm opal acrylic and the floors of the apparatus comprised of a grey Lego® surface. The apparatus was covered by a clear Perspex roof (50 cm × 42 cm). An overhead camera was fixed at a height of 1.0 m above the apparatus. The camera was connected to a DVD-R recorder and a 22-inch screen to allow the experimenter to monitor the animals’ activity within the apparatus. The room was illuminated by diffused lighting originating from a single table lamp equipped with a 50 w lightbulb. White noise was continuously played in the background throughout the experiment to mask extraneous noise. Various junk objects were used, which differed in colour, texture, shape and size. The objects were made of different materials, including ceramic, plastic, rubber, glass, metal and combinations of those materials.

**Fig. 1:**
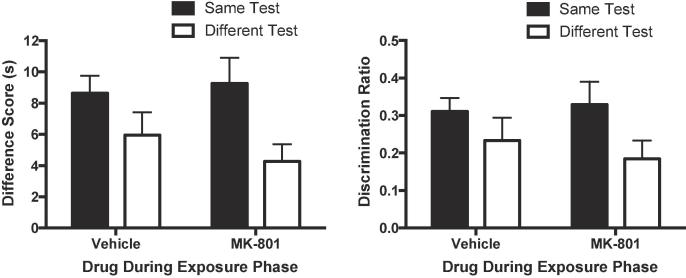
The effect of 0.01 mg/kg MK-801 on object recognition memory. Mice received MK-801 or vehicle prior to the exposure phase. In the test phase the drug-state of mice was either the same or different from that during the exposure phase. Left panel: Performance in the novelty preference test is shown as a difference score (mean time spent exploring the novel objects minus the mean time spent exploring the familiar objects). Scores above zero indicate a novelty preference. Right panel: Performance in the novelty preference test is shown as a discrimination ratio (difference score divided by mean total time spent exploring the objects in the test phase). Error bars indicate SEM.

### 2.4 Procedure

#### 2.4.1 Habituation and pre-training

Mice were exposed to the arena and a selection of objects (that were not used subsequently in the experiments) over a period of seven days and were trained to shuttle between the holding arena and the object arena to consume 0.1 mL sweetened condensed milk (Nestlé UK Ltd., UK) diluted 50% by volume with water. This procedure was used to ensure that mice were motivated to repeatedly enter the holding and object areas. See Chan et al. (2018) for further details.

#### 2.4.2 Exposure and test phase

In both experiments mice were exposed to eight objects during the exposure phase and then were tested 24 h later. In Experiment 1, mice were divided into 4 groups (N = 8 per group): vehicle prior to exposure and test (Vehicle – Same Test); vehicle prior to exposure and MK-801 prior to test (Vehicle – Different Test); MK-801 prior to exposure and test (MK-801 – Same Test); MK-801 prior to exposure and vehicle prior to test (MK-801 – Different Test). In Experiment 2, mice were divided into two groups of 16 animals and received either vehicle or MK-801 prior to both exposure and test. The two groups in Experiment 2 contained equal numbers of the mice assigned to the four groups used in Experiment 1. The experimenter was blind with respect to the group allocation of the mice. The interval between Experiments 1 and 2 was 14 days.

During the exposure phase mice were placed in the holding arena of the apparatus. After one minute the central door opened to allow the mouse to shuttle into the object arena. The mouse was given two minutes to consume the condensed milk and explore a pair of identical objects (e.g., A^1^ and A^2^) located in the top-left and top-right hand corners of the apparatus. At the end of the sample trial, the side doors were opened to allow the mouse to return to the holding arena for one minute, and to consume the condensed milk. While the mouse waited in the holding arena, the experimenter changed the objects in the object arena and the object arena food well was rebaited. After the one minute interval, the central door was opened and the mouse shuttled to the object arena and was now allowed to explore a different pair of identical objects (e.g., B^1^ and B^2^). This procedure was repeated until a mouse had been exposed to eight objects (A-H). Twenty-four hours later mice received the test session that was run in a similar manner to the exposure phase, except that now mice were allowed to explore a familiar object and a novel object on each trial (e.g., A and I). New copies of the familiar objects were used in order to avoid potential odor cues. Across the eight test trials the familiar objects were presented in the same order as the exposure phase. The location of the novel object across trials was equally often left or right for a mouse, but the order of the locations was random with the constraint that it was not in the same location more than twice across consecutive trials. For each pair of novel and familiar objects presented on each trial the identity of the novel object and its location was counterbalanced across mice within each group. The order of the object pairs presented across trials was the same for each mouse. Objects were wiped with 70% Isopropyl alcohol after each trial, and the arena was cleaned with 70% Isopropyl alcohol at the end of each day.

### 2.5 Behavioural data analysis

Object exploration was scored off-line and the scorer was blind with respect to the group allocations of the mice and the identities of the novel objects. Object exploration was defined as the nose of the mouse being directed towards the object at a distance of less than 1 cm. Novelty preference was measured using the difference in the mean duration of exploration of novel and familiar objects (difference score: novel minus familiar) across all eight trials, with positive scores indicating a novelty preference. In addition, difference scores weighted by the mean total object exploration time across trials during the test phase were used to calculate a discrimination ratio with positive ratios indicating a novelty preference (Ennaceur & Delacour, 1988). The path length travelled by mice during the exposure and test phases was used as a measure of locomotor activity. Path lengths were tracked using Ethovision (Noldus, Netherlands). For between group comparisons data were analysed with either multi-factorial ANOVA or unpaired t-tests. One sample t-tests were used to test whether novelty preferences were significantly above chance (i.e., zero for both the difference scores and discrimination ratios). In instances in which we were interested in whether the data provided support for the null hypothesis, Bayes factors (*BF_10_*) were calculated in JASP (JASP Team, 2018) using default priors.

## 3 Results

### 3.1 Experiment 1

During the test phase the novelty preference for mice that received MK-801 during the exposure phase was similar to controls (Fig. 1). However, mice that received the same drug during both the exposure and test phases showed a higher preference than mice that were tested under a drug-state that was different from that in the exposure phase, regardless of whether it was MK-801 or vehicle. A 2 (Drug during exposure: MK-801 or vehicle) by 2 (Drug during test: Same or Different) ANOVA revealed that difference scores (Fig. 1, left panel) were significantly higher when the drug-state at test was the same as exposure than when it was different (F(1, 28) = 8.07, p = 0.008, η_p_^2^ = 0.22, 90% CI [0.04, 0.41]). There was no significant effect of MK-801 during exposure (F < 1), and no significant interaction between factors (F < 1). A similar pattern was found with discrimination ratios (Fig. 1, right panel; same/different effect: F(1, 28) = 4.48, p = 0.043, η_p_^2^ = 0.14, 90% CI [0.002, 0.33]; effect of drug-state at exposure and interaction: F values < 1). One sample t-tests for both difference scores and discrimination ratios confirmed that the novelty preferences for mice tested in the same drug-state as exposure and a state that differed from exposure were significantly above chance (smallest t(15) = 5.51, p < 0.001, η_p_^2^ = 0.67, 90% CI [0.36, 0.78]).

**Fig. 2:**
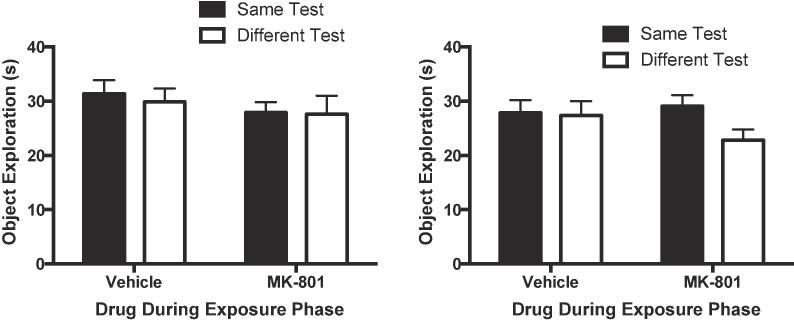
MK-801 (0.01 mg/kg) failed to affect levels object exploration. Left panel: Cumulative duration of object exploration in the exposure phase. Right panel: Cumulative duration of object exploration in the test phase. Error bars indicate SEM.

**Fig. 3:**
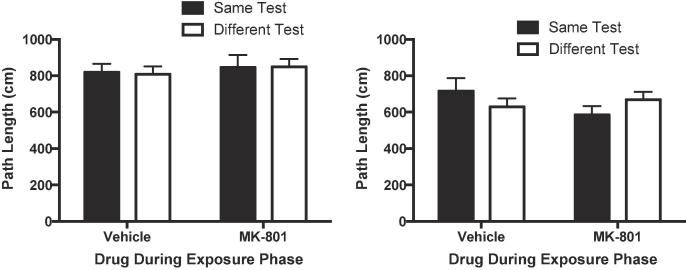
MK-801 (0.01 mg/kg) failed to affect locomotor activity. The distance travelled was used as a measure of locomotor activity. Left panel: Path length in the exposure phase. Right panel: Path length in the test phase. Error bars indicate SEM.

Similar ANOVAs were conducted on the levels of object exploration (Fig. 2) and path lengths (Fig. 3) for both phases. For both measures, there were no significant effects or interactions of factors for either exposure or test phases (object exploration: largest F = 2.26, p = 0.144 η_p_^2^ = 0.07, 90% CI [0.00, 0.25]; path lengths: largest F value = 2.50, p = 0.125, η_p_^2^ = 0.08, 90% CI [0.00, 0.26]).

### 3.2 Experiment 2

**Fig. 4:**
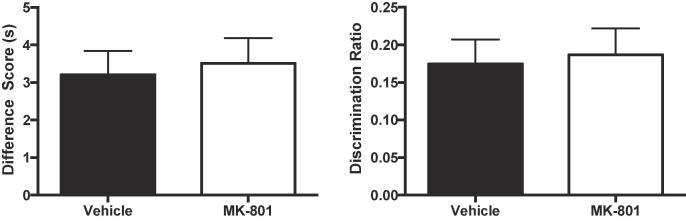
The effect of 0.1 mg/kg MK-801 on object recognition memory. Mice received MK-801 or vehicle prior to both the exposure and test phase. Left panel: Performance in the novelty preference test is shown as a difference score (mean time spent exploring the novel objects minus the mean time spent exploring the familiar objects). Scores above zero indicate a novelty preference. Right panel: Performance in the novelty preference test is shown as a discrimination ratio (difference score divided by mean total time spent exploring the objects in the test phase). Error bars indicate SEM.

During the test phase the novelty preference was similar for both groups (see Fig. 4; difference score: t(30) < 1; discrimination ratio: t(30) < 1). In order to assess whether the data supported the null hypothesis, *BF_10_* was calculated for each measure: difference score, *BF_10_* = 0.351; discrimination ratio, *BF_10_* = 0.345. Therefore, the results are more than 2.8 times more likely under the null hypothesis than under the alternative hypothesis. One sample t-tests for both difference scores and discrimination ratios confirmed that novelty preferences were significantly above chance for both groups (smallest t(15) = 5.06, p < 0.001, η_p_^2^ = 0.46, 90% CI [0.23, 0.60]).

**Fig. 5:**
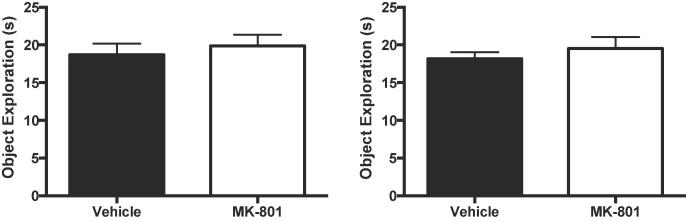
MK-801 (0.1 mg/kg) failed to affect levels object exploration. Left panel: Cumulative duration of object exploration in the exposure phase. Right panel: Cumulative duration of object exploration in the test phase. Error bars indicate SEM.

**Fig. 6:**
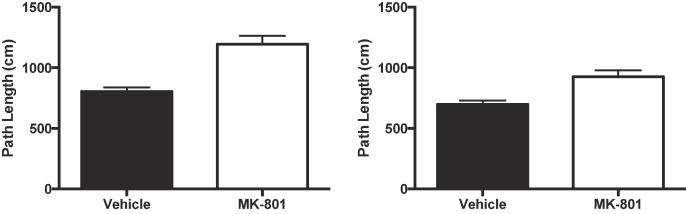
MK-801 (0.1 mg/kg) increased locomotor activity. The distance travelled was used as a measure of locomotor activity. Left panel: Path length in the exposure phase. Right panel: Path length in the test phase. Error bars indicate SEM.

There was no significant effect of MK-801 on object exploration (Fig. 5) during exposure (t < 1) or test (t < 1). MK-801 did significantly increase path lengths (Fig. 6) during the exposure phase (t(30) = 5.07, p < 0.001 η_p_^2^ = 0.46, 90% CI [0.23, 0.60]) and the test phase (t(30) = 3.72, p = 0.001, η_p_^2^ = 0.32, 90% CI [0.10, 0.49]).

## 4 Discussion

The aim of the experiments was to test if MK-801 impairs long-term spontaneous object recognition memory under conditions in which the state-dependency of memory was controlled. When mice received MK-801 prior to both the encoding and test phase there was no significant effect on performance. This was true for a low dose (0.01 mg/kg) and a higher dose (0.1 mg/kg). In contrast, in Experiment 1 it was found that, regardless of the whether mice received vehicle or MK-801 at exposure, if there was a switch in the drug-state between the exposure and test phases then performance was lower than when the states were the same. The impaired performance of object recognition memory when there was a switch in the drug-state between the exposure and test phases did not reflect general changes in object exploration. Levels of object exploration were similar between groups for both the exposure and test phases. The lack of difference between groups when state-dependency was controlled demonstrates that although the low dose of MK-801 clearly led to state-dependent effects, it did not affect encoding or retrieval of memory per se.

The lack of effect of MK-801 on encoding in Experiment 1 is unlikely to be due to the level of the dose. In Experiment 2 mice received a higher dose (0.1 mg/kg) that still did not affect performance under conditions in which the state-dependency of memory was controlled. The high dose did, however, induce hyperactivity, as indicated by an increase in path length, demonstrating that the dose used was able to affect behaviour. Despite this, levels of overall object exploration were not significantly affected by MK-801. It is of course possible that even higher doses may impair memory under conditions in which state-dependency is controlled, but the scope for finding such an effect is likely to be limited due to MK-801 causing ataxia at doses of 0.2 mg/kg (Andine et al., 1999).

The failure to find impaired memory performance with the high dose in Experiment 2 is unlikely to be due to a floor effect. Given that control mice showed performance that was significantly above chance, there was scope to observe reduced performance. Furthermore, the performance of the control mice was comparable to that of controls in other studies using similar procedures that have been sufficient for demonstrating impaired performance (Sanderson et al., 2011). Bayesian analyses provided evidence for the null hypothesis, suggesting that the lack of effect was unlikely to be due to a failure to observe an impairment. Importantly, the lack of effect of the high dose of MK-801 on recognition memory performance was in stark contrast to the effect on locomotor activity, demonstrating that the dose of MK-801 was sufficient to affect behavior despite not impairing recognition memory performance.

The results are in agreement with other studies that have found that MK-801 has state-dependent effects on memory (Ceretta et al., 2008, Flint et al., 2013, Jackson et al., 1992). Due to a lack of effect of MK-801 in our experiments when state-dependency was controlled, conclusions that can be drawn from studies that have not included state-dependent controls are limited. There are, however, studies that have shown MK-801 does affect memory performance when state-dependency is not likely to be an issue. It is of particular importance for the present study that MK-801 impairs object recognition memory when the interval between the exposure and test phases is sufficiently short such that both phases occur during the same exposure to the drug (de Lima et al., 2005, Nilsson et al., 2007, Oh et al., 2017, Olszewski et al., 2012, van der Staay et al., 2011). In these procedures, not only are the exposure and test phases conducted under the same drug-state, but the short retention interval results in short-term memory being assessed. This may suggest that MK-801 impairs short-term object recognition memory, but not long-term object memory. It has been suggested that short-term recognition memory relies on a short-term memory trace that is activated by exploration of an object, whereas long-term recognition memory relies on associative retrieval of memory caused by contextual cues (Robinson and Bonardi, 2015, Sanderson, 2016, Wagner, 1981). Thus it is possible that MK-801 impairs short-term memory activation caused by recent experience of a stimulus, but does not affect associative retrieval of memory. This conclusion is, however, at odds with the findings of Barker et al. (2006), who found that NMDA receptors in the perirhinal cortex, manipulated by the selective NMDA receptor antagonist AP-5, were not necessary for short-term object recognition with a retention interval of 20 mins. This discrepancy may reflect differences in the methods used to manipulate NMDA receptors. It is also possible that NMDA receptors in other brain regions may be responsible for the effects found with systemic administration of MK-801. Barker et al. (2006) did find, however, that AP-5 infused into the perirhinal cortex prior to encoding impaired performance after a 24-hour test interval, but performance during the test was examined in the absence of the drug. Therefore, the impaired performance may reflect a state-dependent effect on memory expression rather than impaired encoding of long-term memory.

The absence of an effect of MK-801 on object recognition memory under conditions in which state-dependency of memory is controlled suggests that the role of NMDA receptors in learning and memory may be more limited than previously thought. While NMDA receptors are necessary for synaptic plasticity within regions implicated in learning and memory it is possible that their role is not always necessarily in encoding or retrieval, but perhaps in other aspects of cognition that affect behaviour. For example, recent evidence has shown the NMDA receptors in the CA1 and dentate gyrus regions of the hippocampus are not necessary for spatial learning, but mice that lack NMDA receptors in those regions fail to express learning in circumstances in which ambiguous cues compete for control of behavior (Bannerman et al., 2012). Therefore, the role of NMDA receptors and NMDA receptor-dependent long-term potentiation may not be in acquisition of memory, but in factors that affect behavioural expression of memory (Bannerman et al., 2014).

Assessment of object recognition memory was optimised in the present study using a procedure that increased the sensitivity of the measure by exposing mice to multiple objects and decreased potential stress by minimizing handling of the mice (Chan et al., 2018). The procedure was adapted from that used by Chan et al. (2018) in order to test performance over a long, 24 h interval and to test the effect of drugs on different stages of the object recognition procedure. While the spontaneous novel object preference procedure is widely used as a measure of memory in rodents (Ennaceur, 2010, Lyon et al., 2012) it often suffers from a lack of sensitivity due to the inherent variability of unconditioned behavior between and within animals (Ameen-Ali et al., 2015, Gaskin et al., 2010, Kinnavane et al., 2015). The results of the present study demonstrate that the multiple-trials procedure allows a measure of memory that is sufficiently sensitive to reveal differences in performance of mice that differ in their current drug-state. Therefore, this procedure is a new viable method for testing the effect of the drugs and other neural manipulations on dissociable components of memory such as encoding and expression.

In conclusion, the results demonstrate that MK-801 fails to impair expression of long-term memory in the object recognition procedure when the state-dependency of memory is controlled. Therefore, MK-801 does not appear to affect encoding of long-term memory, but induces a state that can affect the expression of memory when the drug-state differs between encoding and retrieval of memory.
